# Land and power framework for assessing Ecosystem Essential Area policy

**DOI:** 10.1016/j.mex.2020.101032

**Published:** 2020-08-15

**Authors:** Muhammad Alif K. Sahide, Micah R. Fisher, Emban Ibnurusyd Mas'ud, Wiwik Dharmiasih, Bart Verheijen, Ahmad Maryudi

**Affiliations:** a Forest and Society Research Group, Faculty of Forestry, Universitas Hasanuddin, Makassar, Indonesia; b The University of Hawaii at Manoa, United States; c Universitas Udayana, Bali, Indonesia; d Faculty of Forestry, Universitas Gadjah Mada, Yogyakarta, Indonesia; e Universiteit van Amsterdam, Amsterdam, Netherlands

**Keywords:** Land and power, Ecosystem Essential Area, Anticipating bureaucracy, Multi-stakeholder arrangements

## Abstract

This paper outlines a land and power framework for assessing whether a new voluntary conservation area policy is a return to the classical bureaucratic status quo or anticipates the opportunity to establish new bureaucratic norms. The application of this conceptual framework produces two possibilities. The first possibility is that outcomes are tied to the conventional bureaucratic models of conservation with management regimes that remain unchanged. The second possibility is the anticipation of new management forms, in which goals are not to fulfill the bureaucratic process, but rather, produce adaptive outcomes reflective of the interests of diverse actors engaged in site-specific voluntary conservation initiatives.

• The land and power framework methodology is rooted in an interest-based power framework.
• The framework analyses the land and power inputs for both conservation bureaucracies or actors participating in multi-stakeholder arrangements struggling to achieve their interests and establish their agendas.
• The framework proposes a conceptual framework to assess two possible process outcomes, namely that management regimes will either be tied to the conventional bureaucracy or that actors anticipate new bureaucratic norms that achieve outcomes accommodating their broader interests.

## Graphical abstract

**Figure:**
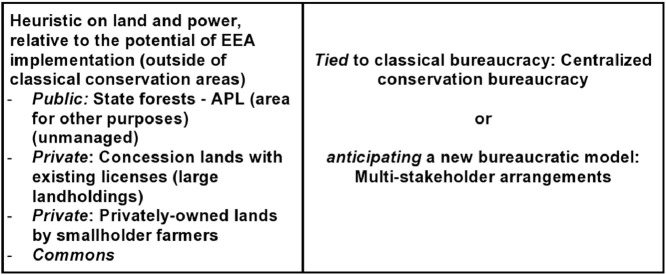

**Table:** Specifications Table

Subject Area:	Environmental Science
More specific subject area:	Forest policy and governance
Method name:	Land and power framework for assessing voluntary conservation development
Name and reference of original method:	Not applicable
Resource availability:	Not applicable

## Method details

Conservation area research has a long tradition that brings together various and evolving research strands. Much of the research is often divided into management approaches and characterized by philosophical differences about the management of land and natural resources. Management approaches are differentiated by their emphasis on the role of the centralized state, the private sector, local communities, and stakeholders, or a combination of the above. More recent formulations have stepped beyond these classical debates to examine the role of power and property in terms of access, exclusion, and authority [1], [2], [3], [4], [5]. Krott et al., [6] further divide power elements into its heuristic parts by examining dominant information, coercion, and incentive/disincentives. The process unfolding amidst Ecosystem Essential Area (EEA) policy formulation is in line with these studies, and also adds an additional dimension of natural resources and decentralization [7] [8], which explicitly raises the issues of downwards and upwards accountability [9]. The EEA policy presents new and slight variations of additional complexity relative to research in these areas [10]. In particular, EEA is a policy instrument that applies conceptually to all land types, borne through private sector voluntary certification schemes, and introduces elements of decentralization that challenge the classical conservation model in Indonesia.

Considering these various policy frameworks, we apply a common formulation among them, particularly highlighting actors/institutions and powers [20], including the different characteristics of power across three different land types (public, private, and commons) to ground our framework. Biodiversity protection on privately-owned lands has been especially problematic [11], where these power contestation may lead to the polycentric form, where many autonomous actors’ interest and influence contested or act in ways that take account of others interest, e.g., conflict of the main objective of land management, implementation preferences, and understanding of policy tools. ‘Unmanaged’ forests under public land designations also have polycentric power dimensions between regulators and land managers, which have created conflict between the state and local communities [12,13]. Among commons lands (namely customary and Indigenous) land, international conservation regimes have elevated the principle of recognizing indigenous rights for conservation area management and Free and Prior Informed Consent. We recognize the complexity and various forms of rules, rights, and responsibilities that shape the commons [14], [15], [16], [17]

Building on Sahide et al. [18], we develop a conceptual framework for assessing EEAs by assigning temporal and thematic elements. The framework first examines the initiation of the policy process. This consists of the overall justification for identifying a high conservation value area, which is not only a technical process but is also contingent upon key actors that can influence the politics of identifying species and conservation priorities at a specific locale. On the one hand, this can take the shape of a palm oil company seeking to obtain private certification (e.g., meet Roundtable for Sustainable Palm Oil certification standards), while on the other, could be based on conservationists using research to identify and promote an area of high conservation value. In line with Howlett [19], the remainder of the framework examines ways for determining the extent to which the form and function of the conservation bureaucracy change.

In this framework (detailed in Table 1), we develop a method to identify to what extent the continued forms of the classical conservation bureaucracy will persist, or whether new conditions will reshape management regimes into a distinct form of bureaucratic-governance and political norms. We define this differentiation as *tied* versus *anticipated*. The tied or anticipated model can either be sustained by the existing bureaucratic model of what we define as the Centralized Conservation Bureaucracy (CCB) or established through new Multi-Stakeholder Arrangements (MSA). Both can maintain the status quo or establish new norms. By *tied*, we mean that conventional models remain unchanged in the form of budget expenditures, expanding mandates for staff and authority, approaches to performance reports responding to national policy and international regimes, and the way that working relations are reinforced with the clients of the bureaucracy. On the other hand, by *anticipated,* we identify whether new mechanisms can take shape. *Anticipating* a new bureaucracy means that there are new policy tools for achieving targeted outputs that reflect more qualitative outcomes tailored to site-specific needs for achieving mutually agreed upon EEA results. In this way, the goals are not to fulfill the bureaucratic process, but rather, to give shape to a more adaptive and responsive approach to assigning and fulfilling targets of the particular EEA initiative at that site. This *anticipated* model, of course, does not suggest that there is no bureaucracy, but rather the heuristic points to ways for identifying a model for new approaches on making the bureaucracy work for output indicators established at a given site.

**Table 1:** Determinant framework: A return to lithe classical bureaucratic status quo versus or anticipating new bureaucratic norms.

Heuristic on land and power, relative to the potential of EEA implementation (outside of classical conservation areas)	*Tied* to classical bureaucracy (T) or *anticipating* a new bureaucratic model (A)		
***Public:* State forests - APL (area for other purposes) (unmanaged)** – National non-conservation bureaucracy or regional bureaucracy owns the land, and have the mandated authority to approve or block an initiative (in both planning and implementation functions) but have not acted upon them. – The conservation bureaucracy serves a function in regulating conservation-oriented initiatives through their understanding of technical regulations and procedures (dominant information) and can apply pressure to regional governments on fulfilling requirements – A select group of CS alliances that have international links and strong relationships with national actors to be able to interpret and reinterpret policy, and convene stakeholders into decision-making processes	**T**	**CCB**	As “unmanaged”^1^ public areas gain attention for potential EEA policy application, CCB justifies activities by invoking dominant information – such as regulations and maps – conducted in partnership with alliances among local government institutions.
		**MSA**	Conversely, MSA arrangements to lead EEA implementation are also likely to seek out formal approvals as a framework for implementation. Implementation will be entrusted to local intermediaries in the form of consultancies that help to interpret policies and justify them under official guidelines. This is particularly likely to occur when quick implementation horizons are demanded that must highlight “results.” Forums are thus created as a way for pointing to broader participation but are careful not to push the boundaries of existing bureaucratic norms.
	**A**	**CCB**	As the CCB will be entering into new territories beyond the previous work of the bureaucracy's mandate, potential innovations are possible. Such innovations can emerge if the overall guiding framework of CCB implementation applies their mandate by achieving essential quality outcomes rather than the classical material outcomes, usually part of the achievement of reporting outcomes. Considering that public land arrangements in EEA will be under a regional government mandate (KPH or regional line agencies), the opportunity for the CCB to facilitate them to lead the process could result in the lead to potential innovations regarding working relationships for meeting project mandates and outcomes. Also, the more detailed understanding among regional governments about the local context and existing management practices also provides them added authority.
		**MSA**	Due to the characteristics of unmanaged public lands, MSA has the opportunity to engage more meaningfully in participatory processes, applying more comprehensive needs assessments, including open public consultation, for identifying potential EEA in public areas. This can lead to new approaches for more responsive institutional mechanisms for establishing management on, for example, the protection of land corridors through the EEA framework
***Private*: Concession lands with existing licenses (large landholdings)** – Concession companies have a strong negotiating stance because there is no strict/mandatory requirement for EEA implementation, and implementation is mostly voluntary. Nevertheless, the conservation bureaucracy relationships with private concessions are influenced by environmental assessment requirements (AMDAL, KLHS, and others). – CS that works with private companies are mostly large players involved in forest certification schemes or with select BINGOs. ***Private*: Privately-owned lands by smallholder farmers** – Smallholders are largely influenced by livelihood decisions or through the facilitation of external actors (CS) that can help to interpret policy, plan, and implement. This could also apply for a network of farmers under community cooperative lands or other means for co-management rights over land that considers ecosystem arrangements (also can be interpreted as commons below).	**T**	**CCB**	CCBs use their power over dominant information to conduct direct and bilateral communication with private concessionaires. On private lands, CCB will not dictate management plans, but they can apply the regulatory requirements as a way to impose their institutional agenda targets.
		**MSA**	Selected and exclusive MSAs, generally overseen by a private corporation, will be involved in fulfilling the formal dimensions of multistakeholder and participatory involvement, taking their cues from the CCB on meeting regulatory requirements. On smallholder lands, the CCB is unlikely to take on a case by case basis, but will engage with strong state or non-state institutions that implement an EEA initiative, and will coordinate through the regional governing bodies.
	**A**	**CCB**	CCB will work to support initiatives on the awareness of private institutions to succeed in their interests to promote and propose EEA initiatives. The CCB therefore, only works for the needs of their partners, developing a more balanced working relationship. The work is seen more voluntarily to provide meaningful support – such as data, indicative maps, etc – for the partner to achieve outcomes of particular EEA interests.
		**MSA**	MSA will play various roles based on the different land-power relations at the concessions. If the concessionaire is closed, then the MSA will play a role as a watchdog. However, if there are potential opportunities to gain buy-in from multiple actors, then the MSA can present a more flexible approach to multi-stakeholder participation similar to those articulated in models of adaptive collaborative management approaches.
***Commons*** – The emergence of compliance considerations related to FPIC (included in HCV) around indigenous lands or forests present opportunities for local leadership in interpreting decisions locally and shaping on implementation arrangements.	**T**	**CCB**	CCB will use the increasing visibility of commons among international regimes (particularly on indigenous lands, HCV, FPIC, etc.) as a way to continue to apply the conventional bureaucratic interpretations. For example, if the formal recognition of indigenous lands is given to the rights of the community, the formal bureaucracy will still seek to apply requirements of developing and implementing forest management plans, in which the successes of which are still being evaluated by the CCB. This is especially true for the category of indigenous forests whereby the MEOFor attest they can reclaim indigenous forest land if the land functions change under indigenous management.
		**MSA**	MSA forums that remain tied to the formal bureaucracy will be shaped by formal power relations. These are often influenced shaped by identity forums that are backed by existing seats of power among the formal bureaucracy.
	**A**	**CCB**	This will be similar to the role that CCB play on private lands, as listed above. This is because the formal institutional interpretations for recognizing the commons (in this case indigenous land rights) have followed the same formal principles in authority conveyed to private ownership.
		**MSA**	MSA will strengthen and facilitate the commons so that meaningful EEA is implemented with the close participation and collaboration of local actors that are rooted in site-specific indigenous and management practices and interpretations.

1 We recognize the notation of unmanaged is a particular bureaucratic terminology, but one that when classified as such, can shape new land management outcomes. CCB: Centralized conservation bureaucracy; MSA = Multi-stakeholder arrangements.

## Declaration of Competing Interest

The authors declare that they have no known competing financial interests or personal relationships that could have appeared to influence the work reported in this paper.
